# Is Hemoglobin Mass at Age 16 a Predictor for National Team Membership at Age 25 in Cross-Country Skiers and Triathletes?

**DOI:** 10.3389/fspor.2021.580486

**Published:** 2021-03-15

**Authors:** Jon Peter Wehrlin, Thomas Steiner

**Affiliations:** Section for Elite Sport, Swiss Federal Institute of Sport, Magglingen, Switzerland

**Keywords:** blood volume, CO-rebreathing, talent identification, adolescents, maturation

## Abstract

We recently measured the development of hemoglobin mass (Hb_mass_) in 10 Swiss national team endurance athletes between ages 16–19. Level of Hb_mass_ at age 16 was an important predictor for Hb_mass_ and endurance performance at age 19. The aim was to determine how many of these young athletes were still members of Swiss national teams (NT) at age 25, how many already terminated their career (TC), and whether Hb_mass_ at ages 16 and 19 was different between the NT and TC group. We measured Hb_mass_ using the optimized carbon monoxide re-breathing technique in 10 high-performing endurance athletes every 0.5 years beginning at age 16 and ending at age 19. At age 25, two athletes were in the NT group and eight athletes in the TC group. Mean absolute, body weight-, and lean body mass (LBM) related Hb_mass_ at age 16 was 833 ± 61 g, 13.7 ± 0.2 g/kg and 14.2 ± 0.2 g/kg LBM in the NT group and 742 ± 83 g, 12.2 ± 0.7 g/kg and 12.8 ± 0.8 g/kg LBM in the TC group. At age 19, Hb_mass_ was 1,042 ± 89 g, 14.6 ± 0.2 g/kg and 15.4 ± 0.2 g/kg LBM in the NT group and 863 ± 109 g, 12.7 ± 1.1 g/kg and 13.5 ± 1.1 g/kg LBM in the TC group. Body weight- and LBM related Hb_mass_ were higher in the NT group than in the TC group at ages 16 and 19 (*p* < 0.05). These results indicate, that Hb_mass_ at ages 16 and 19 possibly could be an important predictor for later national team membership in endurance disciplines.

## Introduction

It is well-documented that elite endurance athletes are characterized by having up to 40% higher levels of total hemoglobin mass (Hb_mass_) than untrained subjects (Kjellberg et al., 1949). Hb_mass_ strongly correlates with maximal oxygen uptake in endurance national team athletes (Schmidt and Prommer, 2008), and there is a strong correlation between Hb_mass_ and endurance performance, even in groups of highly-trained endurance athletes (Hauser et al., 2018; Zelenkova et al., 2019b). Several studies (Wehrlin and Marti, 2006; Wehrlin et al., 2006; Hauser et al., 2016, 2017) have demonstrated that Hb_mass_ in elite athletes increases temporarily with altitude training, but the variation of Hb_mass_ over a training season is minimal (Garvican et al., 2010). In addition, long-term endurance training (without altitude training) in elite athletes does not increase Hb_mass_ (Wehrlin et al., 2016) and Hb_mass_ in elite endurance athletes remains constant over many years (Wehrlin et al., 2016). Therefore, the high Hb_mass_ of elite athletes is more likely to originate from a specific genetic predisposition than from endurance training (Bouchard et al., 1999; Steiner et al., 2019). We previously hypothesized that we could measure Hb_mass_ in endurance athletes at age 16 to identify talent for endurance disciplines. In the first study (Steiner and Wehrlin, 2011), we compared cross-sectional data of Hb_mass_ in cross-country skiers and triathletes at age 16 with Hb_mass_ in national team athletes at ages 19 and 28 elite national team athletes. While the Hb_mass_ measurements at ages 19 and 28 were similarly high, at age 16, athletes had 30% lower values, which did not differ from their age-matched untrained controls (Steiner and Wehrlin, 2011). Therefore, we hypothesized that Hb_mass_ increases with endurance training in adolescents between ages 16 and 19 (Steiner and Wehrlin, 2011). In a follow-up longitudinal study, we continued our Hb_mass_ measurements at age 16 athletes and controls for an additional 3 years (Steiner et al., 2019). Contrary to our hypothesis, there was no difference in the level of body weight related Hb_mass_ between athletes and those in the control group at ages 16 and 19. Furthermore, Hb_mass_ increased to the same extent in the endurance athlete group as in the untrained control group, and there was no correlation between the amount of endurance training and the increase in Hb_mass_ (Steiner et al., 2019). Interestingly, there was a wide range in the Hb_mass_ level at ages 16 and 19, and the level of Hb_mass_ at age 16 was an important predictor for Hb_mass_ and performance at age 19 (Steiner et al., 2019).

However, at age 19, it was not clear which of the athletes were going to be successful in transition to the national team and if a high Hb_mass_ was a possible prerequisite. The aim of this brief report was to analyze how many athletes between ages 16–19 were still members of the Swiss national team at age 25, how many terminated their career, and if Hb_mass_ at ages 16 and 19 was different between these two groups.

## Methods

### Study Design

Six years after the last Hb_mass_ measurement of the athletes at age 19 (Steiner et al., 2019) when the endurance athletes were at age 25, we analyzed which of the 10 cross-country skiers and triathletes were members of the Swiss national team (NT group) and which athletes had terminated their career (TC group). We then retrospectively compared the Hb_mass_ of both groups at ages 16 and 19.

### Subjects

Ten male adolescent endurance athletes (five cross-country skiers and five triathletes) participated in the study. Since no junior national teams exist at age 16 cross-country skiers or triathletes, the inclusion criterion for athletes was a national top 15 overall ranking in either cross-country skiing or triathlon in the season preceding the study period. There was no difference in anthropometric data between the NT and the TC groups.

### Ethics Statement

The study was approved by the Regional Ethic Committee in Berne, Switzerland, and was carried out according to the recommendations of the Helsinki Declaration. All subjects and parents gave their written consent prior to any testing.

### Determination of Hb_mass_

The method is described in detail in the original paper (Steiner et al., 2019). In summary, the athletes and controls inhaled a bolus of pure CO (CO-doses were determined to be 1.2 mL·kg^−1^). Before inhalation, as well as 6 and 8 min after inhalation, capillary blood samples (35 μL) were taken from the participants' earlobe and analyzed for percent carboxyhemoglobin (HbCO) using a diode array spectrophotometer (ABL 800flex, Radiometer A/S, Copenhagen, Denmark). All CO-rebreathing procedures were conducted by the same experienced investigator to avoid inter-tester variability. Our laboratory observed a typical error between 1.1 and 1.4% from duplicate measurements of Hb_mass_ through the described method (Naef et al., 2015).

### Data Analysis

Hb_mass_, anthropometric and training characteristics of the NT and TC groups were presented as the mean ± standard deviation (SD). After passing a normality test (Shapiro-Wilk) and an equal variance test (Brown-Forsyth), differences in Hb_mass_ between the NT and TC groups at ages 16 and 19 were calculated with the Student's *t*-test. Effect sizes were calculated after Cohen (Cohen, 1988).

## Results

**Figure 1:**
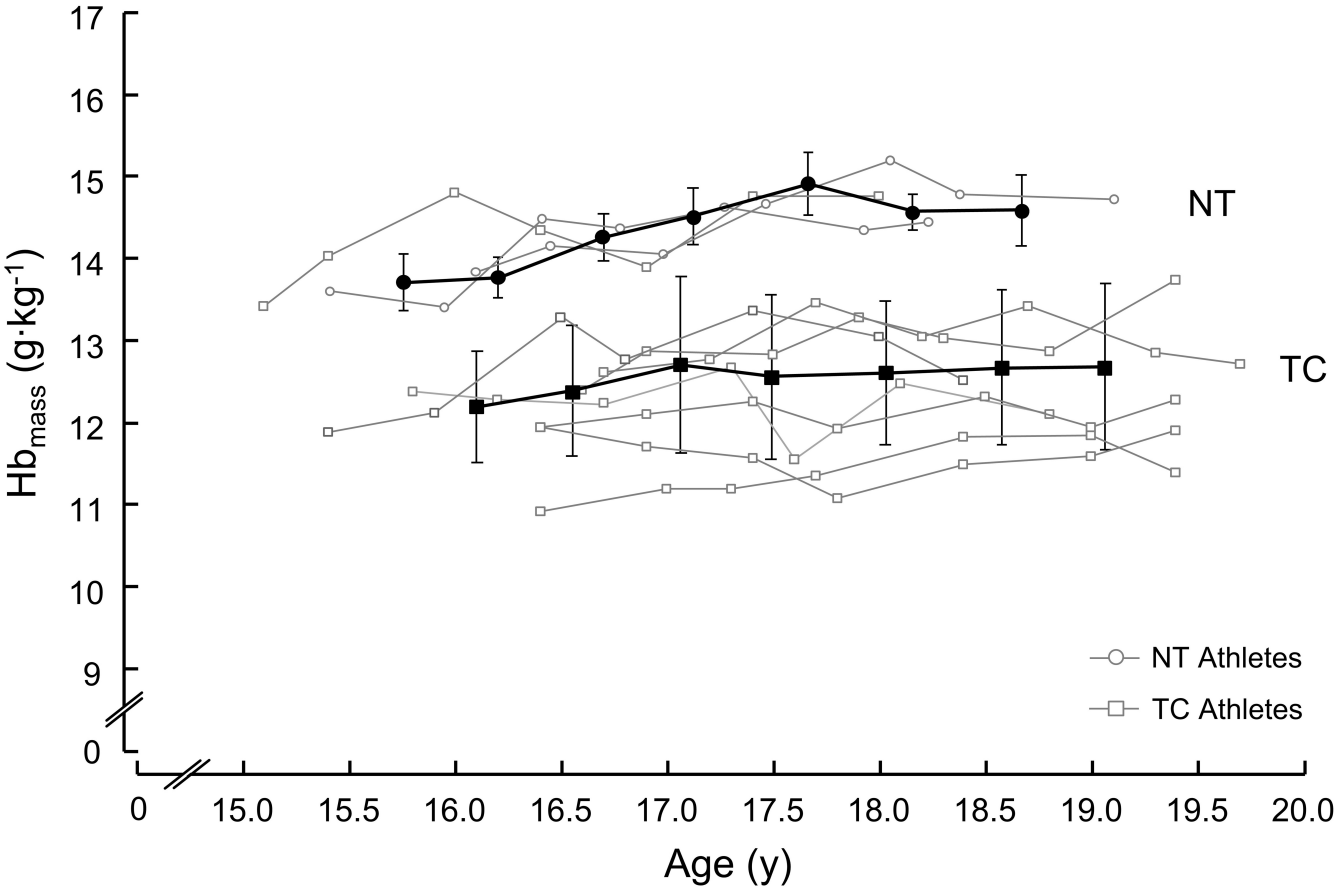
Development of body weight related hemoglobin mass (Hb_mass_) in the national team (NT) group and the terminated career (TC) group during adolescence (ages 16 to 19).

At age 25, two athletes were members of the national team (NT group) and eight athletes had terminated their career (TC group). Mean absolute, body weight-, and lean body mass (LBM) related Hb_mass_ at age 16 were 833 ± 61 g, 13.7 ± 0.2 g/kg and 14.2 ± 0.2 g/kg LBM in the NT group and 742 ± 83 g, 12.2 ± 0.7 g/kg and 12.8 ± 0.8 g/kg LBM in the TC group. At age 19, Hb_mass_ was 1,042 ± 89 g, 14.6 ± 0.2 g/kg and 15.4 ± 0.2 g/kg LBM in the NT group and 863 ± 109 g, 12.7 ± 1.1 g/kg and 13.5 ± 1.1 in the TC group. Body weight- and lean body mass related Hb_mass_ were higher in the NT group than in the TC group at both ages 16 and 19 (*p* < 0.05) and the difference of the groups showed a large effect size (>0.8). Figure 1 shows individual data of all athletes as well as the mean ± SD of the NT and TC groups at and between ages 16 and 19. Table 1 shows anthropometric and training characteristics of both group. There was no difference between groups.

**Table 1:** Characteristics of the NT (*n* = 2) and TC (*n* = 8) group at age 16 and 19 years.

**Group**	**Age (years)**	**Body mass (kg)**	**Height (cm)**	**LBM (kg)**	**VO_2_max (mL · min^−1^)**	**VO_2_max (mL · min^−1^ · kg^−1^)**	**VO_2_max (mL · min^−1^ · kg^−1^ LBM)**	**Endurance training (hours)**	**Total training (hours)**
NT	15.8 ± 0.5	60.8 ± 5.2	178 ± 6	58.6 ± 4.9	4202 ± 108	69.3 ± 4.1	71.8 ± 4.1	7.0 ± 2.8	8.5 ± 3.5
TC	16.1 ± 0.6	60.9 ± 5.8	176 ± 5	57.8 ± 5.0	4037 ± 416	66.3 ± 3.1	69.9 ± 3.6	8.1 ± 1.4	8.7 ± 1.5
NT	18.7 ± 0.6	71.5 ± 7.1	181 ± 7	67.9 ± 6.7	5301 ± 634	74.1 ± 1.6	77.9 ± 1.6	8.2 ± 1.8	9.7 ± 2.3
TC	19.1 ± 0.6	68.0 ± 6.0	180 ± 6	63.9 ± 5.2	4735 ± 378	69.8 ± 4.1	74.2 ± 3.8	8.0 ± 2.3	8.7 ± 2.2

*NT, National team membership at age 25; TC, Terminated career at age 25; LBM, Lean body mass; Values as mean ± SD*.

## Discussion and Conclusion

The main finding of this brief research report is that the mean body weight related Hb_mass_ in the NT group at age 16 was remarkably 1.5 g/kg higher than that in the TC group (1.9 g/kg higher at age 19; Figure 1). Furthermore, body weight related Hb_mass_ values of the NT group reached 13.7 g/kg at age 16 and 14.6 g/kg at age 19. These measurements were very similar to those observed in previous investigations with juniors at age 19 (14.2 g/kg) and national team athletes at age 28 (14.6 g/kg) cross-country skiers and triathletes (Steiner and Wehrlin, 2011). Even though, on average, body weight-related Hb_mass_ increased by ~0.3 g/kg per year between ages 16 and 19, for future elite national team athletes, Hb_mass_ levels of 13.5–14 g/kg at age 16 seem to be a prerequisite to transition to national team at age 25.

We think that a Hb_mass_ level of 14–15 g/kg at age 28 is needed to be a member of a national team in endurance disciplines (Steiner and Wehrlin, 2011). This finding is supported by the fact that Hb_mass_ values from other countries elite male cross-country skiing and triathlon national teams, as well as athletes from other endurance sports, are reported to be about 14–15 g/kg (Heinicke et al., 2001; Wehrlin and Marti, 2006; Gore et al., 2013; Zelenkova et al., 2019a). Mean body weight related Hb_mass_ at age 16 was lower in the NT and TC compared to the Hb_mass_ at ages 19 and 28 (Steiner et al., 2019), which seems to be due to the fact that only two to three athletes per year have the potential to make it to the national team in cross-country skiing and triathlon. Whereas the athletes at ages 19 and 28 (Steiner et al., 2019) consisted of several age cohorts, our athletes at age 16 (NT and TC) consisted of only a 1 year cohort. This means that this group was comprised of physiologically less-talented athletes, which lowered the mean body weight related Hb_mass_.

However, it is important to note that a high body weight related Hb_mass_ is not the only factor related to national team membership at an adult age. While the level of body weight related Hb_mass_ seems to be likely a prerequisite, it is not a guarantee for later national team membership. Other important factors like a high VO_2max_ (and every aspect of oxygen transport), the ability to utilize a high fraction of VO_2max_, a high energy availability, a high anaerobic power, a high efficiency, a high speed capacity and strength in connection with movement specific exercise, play an important role as well (Sandbakk and Holmberg, 2017). It also needs to be emphasized, that this short report suffer from several limitations: The first point is, that we have a very low n (*n* = 2) in the NT group. Results could therefore have been biased by false positive or negative results and other confounding factors. It is also important to mention, that there could be potential differences in the physiological demands between triathlon and cross-country skiers athletes. However, our longitudinal study (Steiner et al., 2019) suggested, that Hb_mass_ at age 16 is an important predictor for Hb_mass_ at age 19. With this additional analysis of national team membership at age 25, we now can further suggest, that a high Hb_mass_ (13.5–14 g/kg) at age 16 could be an important prerequisite for future national team membership at age 28 in endurance disciplines. However, further multi–centric longitudinal studies (merging data from different national training centers) would provide larger sample size for such interesting retrospective talent identification analysis/modeling.

## Data Availability Statement

The raw data supporting the conclusions of this article will be made available by the authors, without undue reservation.

## Ethics Statement

The studies involving human participants were reviewed and approved by Kantonale Ethikkommission Kanton Bern. Written informed consent to participate in this study was provided by the participants' legal guardian/next of kin.

## Author Contributions

JW and TS designed the study, performed the data handling, the interpretation of the data, and the statistical analyses. TS collected the data, contributed to the writing of the manuscript, and designed the figure. JW drafted the manuscript. All authors contributed to the article and approved the submitted version.

## Conflict of Interest

The authors declare that the research was conducted in the absence of any commercial or financial relationships that could be construed as a potential conflict of interest.
